# Genetic diversity of *Fasciola hepatica* in Austria

**DOI:** 10.1007/s00436-020-06633-3

**Published:** 2020-03-03

**Authors:** Christian Husch, Helmut Sattmann, Iveta Haefeli, Heinrich Prosl, Julia Walochnik

**Affiliations:** 1 Institute for Specific Prophylaxis and Tropical Medicine, Medical University Vienna, 1090 Vienna, Austria; 2 Natural History Museum Vienna, 1010 Vienna, Austria; 3 Department of Pathobiology, Institute of Parasitology, Vetmeduni Vienna, 1210 Vienna, Austria

**Keywords:** *Fasciola hepatica*, Digenea, Trematoda, Mitochondrial DNA, Haplotype, Genetic diversity, Austria

## Abstract

The worldwide occurring common liver fluke *Fasciola hepatica* can infect humans and animals and leads to considerable illness and economic loss annually. The aim of this study was to determine the genetic diversity of *F. hepatica* in Austria. In total, 31 adult flukes isolated from cattle from various regions in Austria were investigated for their *cytochrome oxidase subunit 1* (*cox1*) and *nicotinamide dehydrogenase subunit 1* (*nad1*) gene sequences. It was shown that Austrian isolates of *F. hepatica* reveal extensive genetic diversity. To the best of our knowledge, these are the first data on the diversity of *F. hepatica* in Austria.

## Introduction

The parasitic flatworm *Fasciola hepatica* (Trematoda: Fasciolidae), described already by Linnaeus in 1758, has a worldwide distribution (Mas-Coma et al. 2009). It is transmitted by the oral uptake of plants with attached infective metacercariae and can infest a wide range of mammals including humans. Sheep and cattle are the main final hosts, with several hundreds of million infested, causing substantial production losses (Robinson and Dalton 2009). In Europe, infestation rates in sheep and cattle are highly variable, even within countries. In isolated settings, as e.g., on alpine upland farms prevalences can reach over 90% (Ducheyne et al. 2015; Rinaldi et al. 2015). *Galba truncatula* is the main intermediate host in Europe and plays an important role for the distribution of *F. hepatica* (Mas-Coma et al. 2009). Human fasciolosis, caused by *F. hepatica* and *F. gigantica*, is considered a re-emerging neglected disease, with 17 million humans assumed to be infested and 180 million at risk. The disease affects mainly children in poor rural areas, particularly high prevalences of human fasciolosis have been reported for Bolivia, Peru, Ecuador, Egypt, Iran, Vietnam, and China (Mas-Coma et al. 2009). In Austria, the main endemic areas of *F. hepatica* are in the Western federal states (Supperer 1957; Kutzer and Hinaidy 1969; Auer and Aspöck 2014), with domestic as well as game animals being affected. At our institution, we see around 10 human cases of fascioliasis per year, of which, surely some are imported. However, also several autochthonous cases have been recorded, again, mainly from the Western parts of Austria (Auer and Aspöck 2014). Patterns of genetic diversity and population structure may give an insight into the dynamics of dispersal and distribution. The aim of this study was to investigate the diversity of *F. hepatica* in Austria by comparative sequence analyses of the conserved mitochondrial genes *cytochrome oxidase subunit 1* (*cox1*) and *nicotinamide dehydrogenase subunit 1* (*nad1*).

## Material and methods

In total, 31 adult individuals of *F. hepatica* (Fh) isolated from cattle from various regions in Austria were investigated (Table 1). Eight of these samples (Fh1-Fh6 and Fh30-Fh31) were from the collection of the Natural History Museum Vienna and the Franz Berger GmbH & Co KG, respectively. Thirteen samples (Fh7-Fh19) were freshly collected at the abattoir Alpenrind GmbH, Salzburg, and 10 samples (Fh20-Fh29) were provided by the Austrian Agency for Health and Food Safety (AGES).

**Table 1:** Samples of *F. hepatica* with their year of isolation, host and origin and their respective *cox1* and *nad1* subtypes

No.	Year	Host	Origin	Altitude (m a.s.l.)	*cox1* type	*nad1* type
FhAT1	2004	Cattle	Mooslandl, Styria	531	I	I
FhAT2	2013	Cattle	Oberhall, Styria	574	I	I
FhAT3	2013	Cattle	Weissenbach/Enns, Styria	430	VII	I
FhAT4	2013	Cattle	Admont, Styria	640	II	II
FhAT5	2013	Cattle	Johnsbach, Styria	853	II	II
FhAT6	2013	Cattle	Weißenbach/Enns, Styria	430	IX	I
FhAT7	2015	Cattle	Hopfgarten im Brixental, Tyrol	622	II	II
FhAT8	2015	Cattle	Itter, Tyrol	703	II	II
FhAT9	2015	Cattle	Westendorf, Tyrol	783	IV	II
FhAT10	2015	Cattle	Eugendorf, Salzburg	560	III	II
FhAT11*	2015	Cattle	Seekirchen/Wallersee, Salzburg	512	VIII	I
FhAT12*	2015	Cattle	Seekirchen/Wallersee, Salzburg	512	I	I
FhAT13	2015	Cattle	Eugendorf, Salzburg	560	II	II
FhAT14	2015	Cattle	Zell am Ziller, Tyrol	575	I	I
FhAT15	2015	Cattle	Feldkirchen, Carinthia	554	I	I
FhAT16	2015	Cattle	Ottenschlag, Lower Austria	849	V	I
FhAT17	2015	Cattle	Ebbs, Tyrol	475	II	II
FhAT18	2015	Cattle	Söll, Tyrol	703	I	IV
FhAT19	2015	Cattle	Maishofen, Salzburg	768	I	I
FhAT20	2015	Cattle	Kitzbühel, Tyrol	762	I	I
FhAT21	2015	Cattle	Leutasch, Tyrol	1136	VI	I
FhAT22	2015	Cattle	Kössen, Tyrol	588	I	I
FhAT23	2015	Cattle	Angerberg, Tyrol	650	II	V
FhAT24	2015	Cattle	Angerberg, Tyrol	650	III	III
FhAT25	2015	Cattle	Angerberg, Tyrol	650	I	I
FhAT26	2015	Cattle	Mühlbachl, Tyrol	995	I	I
FhAT27	2015	Cattle	Neustift, Tyrol	994	I	I
FhAT28	2015	Cattle	Ellbögen, Tyrol	1070	I	I
FhAT29	2015	Cattle	Innsbruck, Tyrol	574	I	I
FhAT30	2015	Cattle	St. Aegyd, Lower Austria	588	X	I
FhAT31	2015	Cattle	Schwarzenbach, Lower Austria	510	I	I

*flukes deriving from the same cow

### PCR

For molecular analysis, 20–25 mg tissue were cut into small pieces with a scalpel. To avoid inclusion of foreign sperm, the tissue was taken from the apical zone of the flukes (Moazeni et al. 2012). Whole-cell DNA was isolated using the QIAamp DNA Mini Kit (QIAGEN, Vienna). The DNA yield was measured with a NanoDrop ND-1000 spectrophotometer (Peqlab, Erlangen, Germany).

Fragments of the mitochondrial DNA (mtDNA) genes *cox1* and *nad1* were amplified using the forward primer for *cox1* (5´-TTGGTTTTTTGGGCATCCT-3′) from Itagaki and Tsutsumi (1998) and the reverse primer for *cox1* (5´-AGGCCACCACCAAATAAAAGA-3′) and the forward (5´-TATGTTTTGTACGGGATGAG-3′) and reverse primer (5′- AACAACCCCAACCAACACTTA-3′) for *nad1* from Semyenova et al. (2006). The mitochondrial genome of *F. hepatica* has a length of 14,461 bp, the *cox1* gene is located between bp 6,871 and 8,402, and the *nad1* gene is located between bp 5,176 and 6,078. The expected size of the *cox1* amplicon is ~ 500 bp and the one of the *nad1* amplicon ~ 420 bp. The primers were ordered from Microsynth AG (Balgach, Switzerland).

PCR and sequencing were performed as described previously (Husch et al. 2017). In brief, PCR included 15 min of initial denaturation with 95 °C and 30 cycles with 95 °C for 1 min, 56 °C for 2 min, 72 °C for 3 min, and a final extension at 72 °C for 7 min. Bands were visualized under UV light in 2% agarose gels and extracted from the gels using the QIAquick® Gel Extraction Kit (QIAGEN, Vienna). Sequencing PCRs were run with an initial denaturation at 96 °C for 30 s followed by 40 cycles of 96 °C for 10 s, 50 °C for 5 s, and 60 °C for 4 min. Sequences were obtained from both strands in two independent set-ups by direct sequencing with an automated ABI PRISM 310 Sequencer (PE Applied Biosystems, Langen, Germany) and assembled to consensus sequences using GeneDoc (Nicholas et al. 1997).

### Sequence analyses

All consensus sequences were blasted against the reference sequences of *F. hepatica* available in GenBank by using BLAST (Altschul et al. 1990). For subtyping, multiple alignments of all isolates were performed with ClustalX (Thompson et al. 1997). Alignments were manually edited with GeneDoc (Nicholas et al. 1997) to exclude primer regions and to calculate identity scores. All subtypes were compared to the reference sequences of *F. hepatica* available in GenBank. Identities were evaluated separately for *cox1* and *nad1*. Haplotype analyses were performed using PopART 1.7 (Leigh and Bryant 2015). For haplotype analyses, alignments were trimmed to the lengths of the available reference sequences in GenBank, i.e., to 310 bp for *cox1* (AF216697, AP017707, GQ121276, GQ231549, GQ231550, GQ231551, GU112454, JF824670, JF824674, KJ200621, KU555842, KX470584, KX856338, MH561925, MH681796, MK212142, MN006838, X15613) and to 387 bp for *nad1* (AF216697, AP017707, KR422393, KR422396, KT893736, KU946972, LC076257, MF287675, X15613), and TCS networks were obtained.

Voucher specimens of all samples were deposited in the Natural History Museum of Vienna, Austria. All sequence data were submitted to GenBank and are available under the following accession numbers: MN507437-MN507467 (*cox1*) and MN507406- MN507436 (*nad1*).

## Results and discussion

This is the first study on the diversity of *F. hepatica* in Austria. Cattle positive for *F. hepatica* were from 475 to 1136 m above sea level and typically in their older age, between 3 and 8 years old. All flukes isolated were adult individuals; the sizes ranged from 2.5 to 3.2 cm. The *cox1* fragments had a length of 496 bp in all isolates, and the *nad1* fragments had a length of 416–417 bp, depending on the isolate. Altogether, 10 haplotypes were found for *cox1* and 5 haplotypes for *nad1* (Table 1); however, the *nad1* fragment investigated was also shorter. Nevertheless, the diversity level was higher in *nad1* compared to *cox1*, as has been found by others (Semyenova et al. 2006). The differences between the *cox1* haplotypes were between 1 and 5 bp (Table 2) and between the *nad1* haplotypes were between 1 and 6 bp (Table 3).

**Table 2:** Base pair differences between the *cox1* subtypes

	I	II	III	IV	V	VI	VII	VIII	IX	X
I	0									
II	4	0								
III	3	1	0							
IV	3	1	2	0						
V	1	5	4	4	0					
VI	1	5	4	4	2	0				
VII	1	3	4	2	2	2	0			
VIII	1	3	2	2	2	2	2	0		
IX	2	2	1	1	3	3	3	1	0	
X	2	4	5	3	3	3	1	3	4	0

**Table 3:** Base pair differences between the *nad1* subtypes

	I	II	III	IV	V
I	0				
II	4	0			
III	3	1	0		
IV	1	5	4	0	
V	2	2	4	6	0

**Fig. 1:**
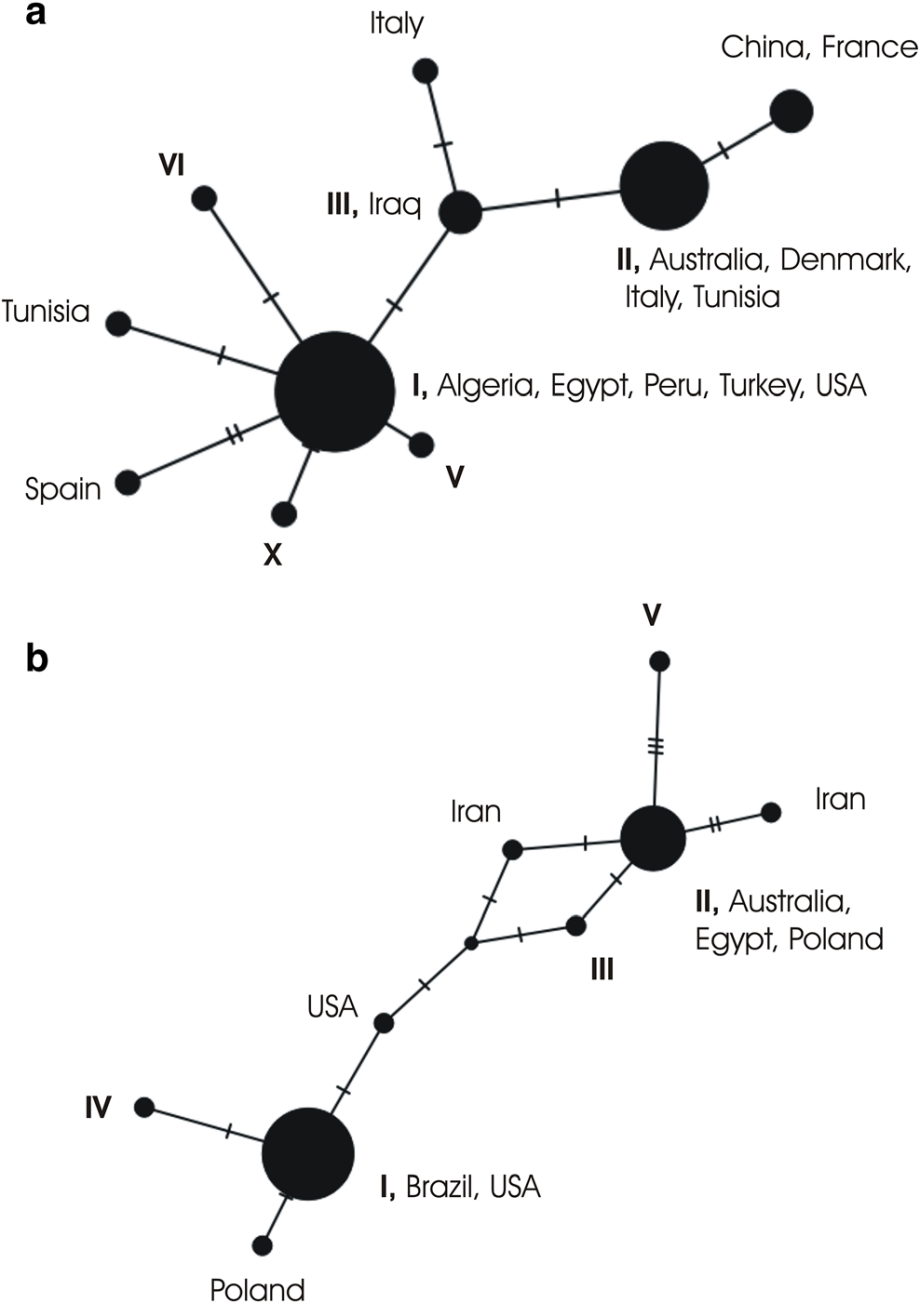
Haplotype networks. **a** based on the *cox1* gene (310 bp). **b** based on the *nad1* gene (387 bp); including all samples from Austria together with the respective reference sequences of *F. hepatica* from GenBank. Note: As sequences had to be trimmed to the lengths of the respective available reference sequences, not all haplotypes detected among the Austrian samples are represented by individual nodes

The investigated 31 individuals of *F. hepatica* clustered into three major groups, termed subtypes I–III, and several more subtypes are only represented by one isolate each. Overall, the typing was rather consistent between *cox1* and *nad1* (Table 1). The most common subtype was subtype I, represented by 15 isolates for *cox1* and by 20 isolates for *nad1*, and being also the only subtype found in all federal states of Austria investigated. This subtype I (Cox1-I/Nad1-I) reveals a 100% identity (*cox1* 496/496 bp, *nad1* 417/417) to one of the reference strains for the mitochondrial genome (AP017707) and 1 bp difference in each gene (fragment) to another mitochondrial genome strain (X15613; *cox1* 495/496 bp, *nad1* 416/417), both isolates from the USA. Moreover, it also shows 100% identities with strains from various countries all over the world, of which however, only shorter fragments or only one fragment is available, e.g., from Turkey (GQ121276), South Africa (KT182303), Algeria (MK212144), and Niger (FJ469984). Subtype II (Cox1-II/Nad1-II) shows a 100% identity to strain Geelong isolated in Australia (AF216697; *cox1* 496/496, *nad1* 417/417) and also to partly shorter fragments from strains from, e.g., Tunisia (GQ231550), South Africa (KT182261), Italy (JF824674), Denmark (MH561925), Poland (KR422395), Egypt (LC076257), and Iran (GQ175362). Subtype III (Cox1-III/Nad1-III) has 1–3 bp differences per gene, depending on the gene, to the three genome reference strains mentioned above (e.g., X15613; *cox1* 494/496; *nad1* 415/417) and again 100% identities to various shorter fragments from strains from all over the world. Haplotype relationships are given in Fig. 1. Interestingly, the flukes Fh11 and Fh12, which derived from the same cow, had the same *nad1* haplotype but a different *cox1* haplotype. Samples Fh10 and Fh13, deriving from two different individuals but from the same farm, also had the same *nad1* haplotype but a different *cox1* haplotype. It has been shown previously that a host can hold up to 10 different mitochondrial haplotypes (Elliott et al. 2014; Walker et al. 2007).

Alasaad et al. (2007) and Semyenova et al. (2006) examined the diversity of *F. hepatica* for several European and non-European countries and also demonstrated high similarities between strains from different continents, which they assume to be mainly due to livestock trafficking. However, depending on the regional setting, the genetic diversity differs dramatically between countries, determined by geography and landscape, on the one hand, but, of course, also on by the predominant type of farming. For example, on the Italian Island of Sardinia, Farjallah et al. (2013) only found three *cox1* haplotypes and 5 *nad1* haplotypes in 66 isolates from sheep and cattle. Walker et al. (2007) investigated 221 flukes from seven different locations in Ireland and found 18 composite haplotypes for *cox3*/*nad4* and 11 composite haplotypes for *cox1*/*rrna.* In a study from the Netherlands, 92 *cox3* haplotypes were detected among 422 flukes isolated from 20 cattle from only two farms (Walker et al. 2011). Finally, also the utilization of anthelmintic drugs might have a significant impact on the genetic diversity of flukes (Walker et al. 2007, 2011; Elliott et al. 2014).

In the current study, the highest diversity was found in the Tyrol, with 5 of the 10 *cox1* subtypes and all of the 5 *nad1* subtypes. However, the Tyrol was also represented by the most isolates. It is considered the main endemic region for *F. hepatica* in Austria (Auer and Aspöck 2014). This might also be attributed to the prevailing husbandry conditions. The Western Austrian states, particularly the Tyrol, Vorarlberg, and Salzburg, are alpine regions characterized by small farms, while the Eastern Austrian states, particularly Lower Austria, are characterized by flat land with large agricultural holdings. Also, precipitation rates are much higher in Western Austria. In this study, all flukes investigated were from cattle; however, sequences from reference strains with highest identities to our isolates were from different host species (cattle, sheep, or humans) corroborating the known low host specificity of *F. hepatica*. Today, *F. hepatica* is the most widely distributed vector-borne parasitic disease (Mas-Coma et al. 2009). A recent study from Armenia suggests that the species might have evolved in temperate Eurasia, where particularly high genetic diversity is found (Aghayan et al. 2019).

In conclusion, *F. hepatica* was isolated from cattle from up to 1136 m above sea level and typically from older aged animals (>3 years). Altogether, 10 haplotypes for *cox1* and 5 for *nad1* were detected, most of them with a 100% identity to isolates from all over the world. Most positive cattle were from the Tyrol, where we also saw the highest genetic diversity.
